# Validation of DAB2IP methylation and its relative significance in predicting outcome in renal cell carcinoma

**DOI:** 10.18632/oncotarget.8971

**Published:** 2016-04-25

**Authors:** Zong-Ren Wang, Jin-Huan Wei, Jian-Cheng Zhou, Ahmed Haddad, Liang-Yun Zhao, Payal Kapur, Kai-Jie Wu, Bin Wang, Yan-Hong Yu, Bing Liao, Da-Lin He, Wei Chen, Vitaly Margulis, Jer-Tsong Hsieh, Jun-Hang Luo

**Affiliations:** ^1^ Department of Urology, First Affiliated Hospital, Sun Yat-sen University, Guangdong, China; ^2^ Department of Urology, University of Texas Southwestern Medical Center at Dallas, Dallas, TX, USA; ^3^ Department of Urology, Shaanxi Provincial People's Hospital, Shaanxi, China; ^4^ Department of Urology, Affiliated Hospital of Kunming University of Science and Technology, Yunnan, China; ^5^ Department of Pathology, University of Texas Southwestern Medical Center at Dallas, Dallas, TX, USA; ^6^ Department of Urology, First Affiliated Hospital of Xi'an Jiaotong University, Shaanxi, China; ^7^ Department of Pathology, First Affiliated Hospital, Sun Yat-sen University, Guangdong, China

**Keywords:** DAB2IP, DNA methylation, intratumour heterogeneity, prognosis, renal cell carcinoma

## Abstract

We have recently reported tumor suppressive role of DAB2IP in RCC development. In this study, We identified one CpG methylation biomarker (DAB2IP CpG1) located UTSS of DAB2IP that was associated with poor overall survival in a cohort of 318 ccRCC patients from the Cancer Genome Atlas (TCGA). We further validated the prognostic accuracy of DAB2IP CpG methylation by pyrosequencing quantitative methylation assay in 224 ccRCC patients from multiple Chinese centers (MCHC set), and 239 patients from University of Texas Southwestern Medical Center at Dallas (UTSW set) by using FFPE samples. DAB2IP CpG1 can predict the overall survival of patients in TCGA, MCHC, and UTSW sets independent of patient age, Fuhrman grade and TNM stage (all *p*<0.05). DAB2IP CpG1 successfully categorized patients into high-risk and low-risk groups with significant differences of clinical outcome in respective clinical subsets, regardless of age, sex, grade, stage, or race (HR: 1.63-7.83; all *p*<0.05). The detection of DAB2IP CpG1 methylation was minimally affected by ITH in ccRCC. DAB2IP mRNA expression was regulated by DNA methylation *in vitro*. DAB2IP CpG1 methylation is a practical and repeatable biomarker for ccRCC, which can provide prognostic value that complements the current staging system.

## INTRODUCTION

More than 270,000 people are expected to be diagnosed with kidney cancer every year worldwide [1]. Clear cell RCC (ccRCC) is the most common subtype of kidney cancer, accounting for approximately 80–90% of cases [2]. Although extensive effort has been devoted to identifying molecular biomarkers for ccRCC, there are few validated markers that aid disease prognosis, and none are used routinely in clinical practice [3–5].

We previously employed a yeast two-hybrid system to identify DAB2IP, a Ras GTPase-activating protein (GAP) that interacts with the N-terminal domain of DOC-2/DAB2 [6]. In general, the epigenetic modification (such as DNA methylation) of DAB2IP gene promoter is responsible for DAB2IP expression [7]. Clinically, DAB2IP gene hypermethylation is correlated with the development of many cancer types. Indeed, DAB2IP functions as a potent tumor suppressor by inhibiting tumor cells proliferation, epithelial-to-mesenchymal transition leading to cancer metastasis, and the appearance of cancer stem cell. Also, loss of DAB2IP in prostate cancer cells acquires their radio- or chemo-therapy resistance [8–12]. Recently, we reported that loss of DAB2IP enhances the malignant transformation of RCC and ccRCC resistance to targeted therapeutics [13].

In this study, we identified one CpG methylation biomarker located at upstream of the transcription start site (UTSS) of DAB2IP (DAB2IP CpG1) that predicted survival in ccRCC patients from The Cancer Genome Atlas (TCGA) study. We validated the prognostic accuracy of DAB2IP CpG1 methylation by pyrosequencing quantitative methylation assay in a population of ccRCC patients from multiple Chinese centers, and in an external validation patient group from University of Texas Southwestern Medical Center at Dallas by using FFPE samples. The impact of intratumor heterogeneity (ITH) on DAB2IP methylation in ccRCC was also assessed in this study.

## RESULTS

### DAB2IP CpG1 methylation correlates with overall survival of ccRCC patients from TCGA

**Figure 1:**
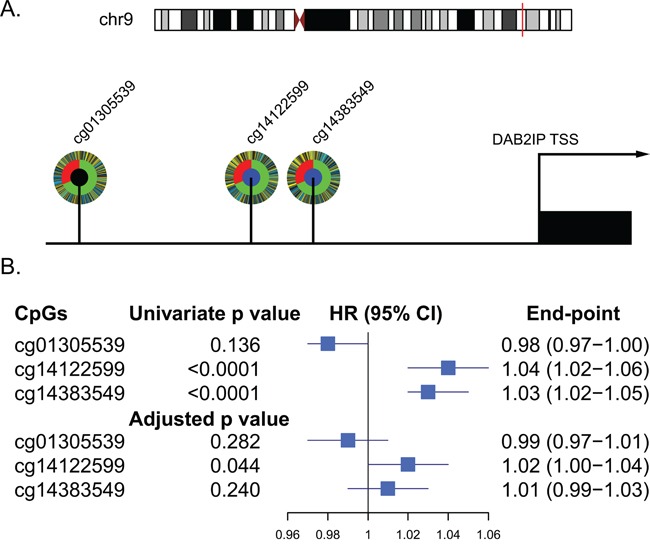
Relationship between CpGs located in the UTSS (upstream of the transcription start site) of DAB2IP and ccRCC patients survival in TCGA set (*n*=318) **A.** Schematic diagram of the three CpGs located in the UTSS (upstream of the transcription start site) of DAB2IP and their corelation with the survival of ccRCC patients. Each CpG is depicted as a multi-ring circle with various levels of data, plotted such that each ‘spoke’ in the ring represents a single patient sample (same sample ordering for all CpGs). The outer ring, inferred levels of CpG methylation (yellow, higher methylation level; blue, lower methylation level); The middle ring, survival status of the patients (red, dead; green, alive); The inner ring, correlation of CpG methylation with patients survival (blue, significantly positive correlation; black, no significant correlation). **B.** Forest plot showed results of the univariate and multivariate analysis of the three CpGs with the overall survival of the patients.

Using the annotations provided by Illumina for the HumanMethylation450 platform, three probes located in UTSS of DAB2IP (cg14122599, cg14383549 and cg01305539) were analyzed in this study (Supplementary Table 1). We investigated the relationship between the three UTSS CpG methylation sites and patient prognosis of ccRCC in the TCGA set. In univariate survival analyses, both cg14122599 and cg14383549 had a significant impact on patient survival (*p*<0.0001). After adjusting for clinical variables (age, Fuhrman grade, pT and pM stages), cg14122599 was an independent prognostic factor for overall survival of ccRCC patients (hazard ratio [HR] 1.02; 95% CI, 1.00-1.04; *p*=0.044) (Figure 1). We refer to cg14122599 as DAB2IP CpG1 in the manuscript.

### Validate DAB2IP CpG1 methylation on ccRCC patient survival by MCHC and UTSW sets

**Figure 2:**
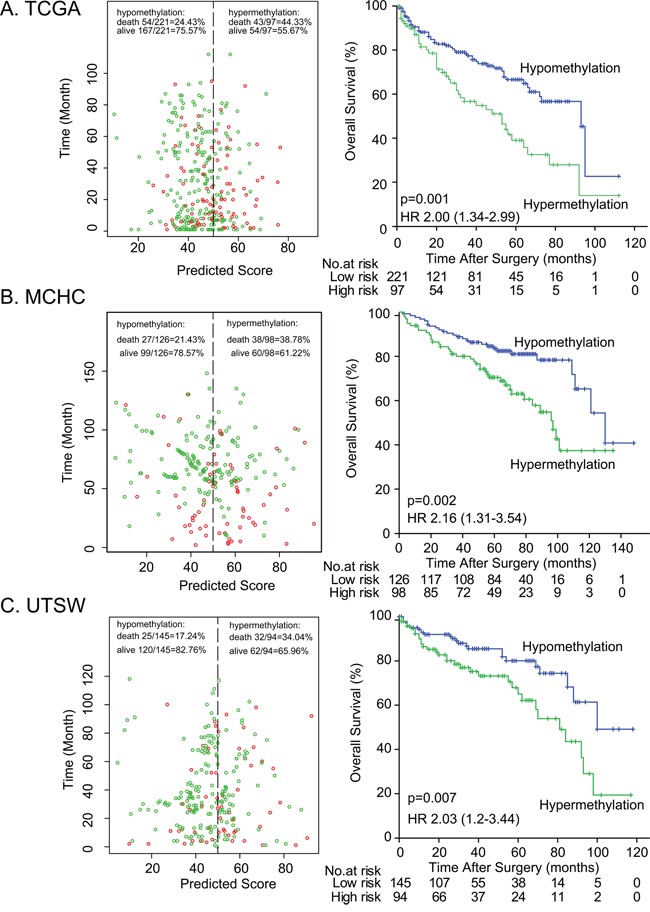
Risk classification by DAB2IP CpG1 and Kaplan-Meier survival in the three different sets **A.** MCHC set, **B.** UTSW set, and **C.** TCGA set. Left panel: The scatter dot plot showed the survival of the patients (green, alive; red, dead). The patients were divided into low-risk and high-risk groups using the cut-off value of 50%. Right panel: Kaplan-Meier survival analysis for the patients. *p* values were calculated using the log-rank test.

**Figure 3:**
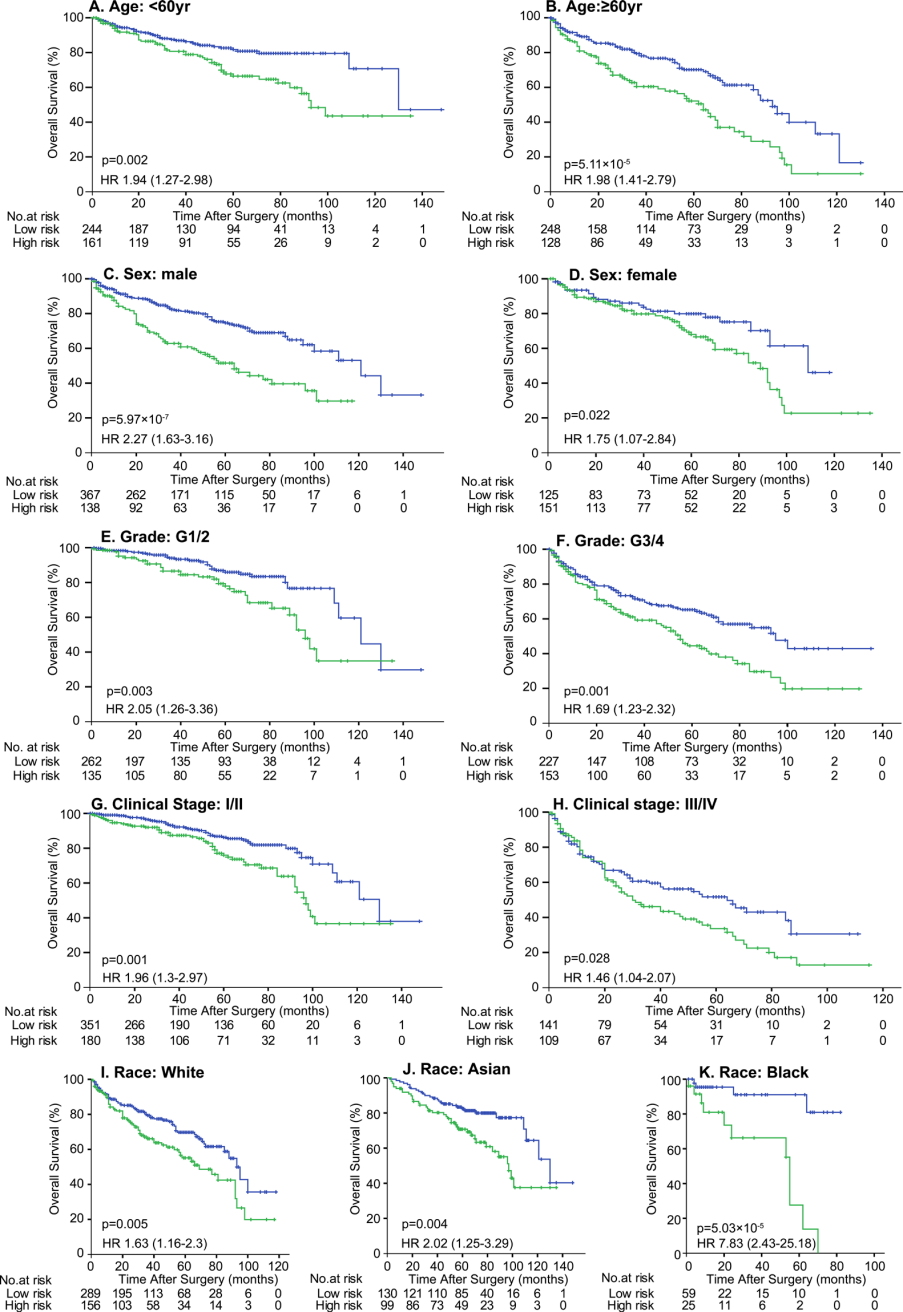
Kaplan-Meier survival analysis of DAB2IP CpG1 methylation in subsets of 781 patients stratified by clinicopathological risk factors Patients were divided into 2 groups based on DAP2IP methylation: High risk group, >50% methylation (green line) and low risk group (blue line), ≤50% methylation. *p* values were calculated using the log-rank test.

To validate the impact of DAB2IP CpG1 methylation on patient survival, we performed pyrosequencing to quantify methylation at DAB2IP CpG1 in the MCHC and UTSW sets. The detailed clinicopathological characteristics of the TCGA, MCHC, and UTSW sets were summarized in Table 1. Overall, the median follow-up was 38 months (IQR 14–67), and 219 (28.0%) of 781 patients died during the follow-up period. Univariate Cox regression analysis confirmed DAB2IP CpG1 methylation had a significant impact on overall survival of ccRCC patients (Table 2). After multivariable adjustment by clinicopathological variables (age, nuclear grade and TNM), DAB2IP CpG1 remained an independent factor (all *p*<0.05, Table 3). Patients in the three sets were divided into high-risk or low-risk groups, using the cut-off value of 50.0% methylation. Compared with patients in low-risk group, patients in the high-risk group had shorter overall survival (HR, 2.00–2.16; log-rank test *p*, 0.007–0.001, Figure 2). Additionally, survival analysis was performed with regard to DAB2IP CpG1 methylation in subsets of patients with different clinicopathological variables. When stratified by clinicopathological variables (sex, age, race, Fuhrman grade, clinical stage), DAB2IP CpG1 was still a clinically and statistically significant prognostic marker (Figure 3, Supplementary Figures 1, 2).

**Table 1:** Baseline characteristics of patients in the three sets

Characteristic	TCGA set (*n*=318)	MCHC set (*n*=224)	UTSW set (*n*=239)
**Age — no. (%)**			
<60	137 (43.1)	142 (63.4)	126 (52.7)
≥60	181 (56.9)	82 (36.6)	113 (47.3)
**Sex — no. (%)**			
Male	204 (64.2)	152 (67.9)	149 (62.3)
Female	114 (35.8)	72 (32.1)	90 (37.7)
**Race — no. (%)**			
Asian	1 (0.3)	224 (100)	4 (1.7)
White	266 (83.6)	0 (0)	179 (74.9)
Black	48 (15.1)	0 (0)	36 (15.0)
Not Available	3 (1.0)	0 (0)	20 (8.4)
**Grade — no. (%)**			
G1	9 (2.8)	10 (4.5)	9 (3.8)
G2	133 (41.8)	108 (48.2)	128 (53.5)
G3	122 (38.4)	76 (33.9)	75 (31.4)
G4	50 (15.7)	30 (13.4)	27 (11.3)
Not Available	4 (1.3)	0	0
**Staging (TNM) — no. (%)**			
T1	158 (49.7)	133 (59.4)	154 (64.4)
T2	41 (12.9)	43 (19.2)	30 (12.6)
T3	111 (34.9)	45 (20.1)	50 (20.9)
T4	8 (2.5)	3 (1.3)	5 (2.1)
**Nodes — no. (%)**			
Node - (N0)	132 (41.5)	210 (93.7)	222 (92.9)
Node + (N1)	9 (2.8)	14 (6.3)	17 (7.1)
Node unknown (NX)	177 (55.7)	0 (0)	0 (0)
**Metastasis — no. (%)**			
Mets - (M0)	234 (73.6)	220 (98.2)	219 (91.6)
Mets + (M1)	53 (16.7)	4 (1.8)	20 (8.4)
Metastasis unknown (MX)	31 (9.7)	0	0
**Clinical stage — no. (%)**			
Stage I	155 (48.8)	128 (57.1)	153 (64.0)
Stage II	31 (9.7)	39 (17.4)	25 (10.5)
Stage III	75 (23.6)	42 (18.8)	39 (16.3)
Stage IV	57 (17.9)	15 (6.7)	22 (9.2)

**Table 2:** Univariate association of DAB2IP CpG1 methylation with overall survival in the three sets

Parameters	TCGA set		MCHC set		UTSW set	
	*P* value	HR (95%CI)	*P* value	HR (95%CI)	*P* value	HR (95%CI)
Age (year)	0.003	1.03 (1.01-1.04)	0.027	1.02 (1.00-1.05)	0.005	1.03 (1.01-1.06)
Sex (male vs female)	0.755	0.94 (0.61-1.43)	0.758	0.92 (0.54-1.56)	0.359	0.78 (0.45-1.33)
T (T1 vs T2 vs T3 vs T4)	<0.0001	2.26 (1.78-2.86)	0.001	1.59 (1.20-2.10)	<0.0001	2.26 (1.70-3.01)
N (N0 vs N1)	0.072	2.35 (0.93-5.99)	<0.0001	5.05 (2.38-10.75)	<0.0001	7.92 (4.09-15.33)
M (M0 vs M1)	<0.0001	4.50 (2.98-6.79)	0.0003	8.86 (2.68-29.28)	<0.0001	4.95 (2.63-9.34)
Grade (G1 vs G2 vs G3 vs G4)	<0.0001	2.84 (2.12-3.80)	0.002	1.63 (1.20-2.20)	<0.0001	2.80 (1.97-3.96)
DAB2IP (methylation level)	<0.0001	1.04 (1.02-1.06)	0.001	1.02 (1.01-1.04)	0.007	1.02 (1.01-1.04)

**Table 3:** Multivariate Cox regression analysis of DAB2IP CpG1 with overall survival in the three sets

Parameters	TCGA set		MCHC set		UTSW set	
	*P* value	HR (95%CI)	*P* value	HR (95%CI)	*P* value	HR (95%CI)
Age (year)	0.004	1.03 (1.01-1.05)	0.004	1.03 (1.01-1.05)	0.003	1.04 (1.01-1.06)
T (T1 vs T2 vs T3 vs T4)	0.008	1.45 (1.10-1.90)	0.226	1.22 (0.88-1.68)	0.093	1.40 (0.95-2.07)
N (N0 vs N1)	-	-	<0.0001	5.05 (2.29-11.13)	0.065	2.17 (0.95-4.93)
M (M0 vs M1)	0.0001	2.52 (1.57-4.04)	0.0001	13.38 (3.53-50.77)	0.035	2.28 (1.06-4.88)
Grade (G1 vs G2 vs G3 vs G4)	0.002	1.69 (1.22-2.34)	0.139	1.29 (0.92-1.79)	0.013	1.74 (1.13-2.70)
DAB2IP (methylation level)	0.044	1.02 (1.00-1.04)	0.005	1.02 (1.01-1.04)	0.001	1.04 (1.02-1.06)

### Impact of ITH on DAB2IP CpG1 methylation in ccRCC

**Figure 4:**
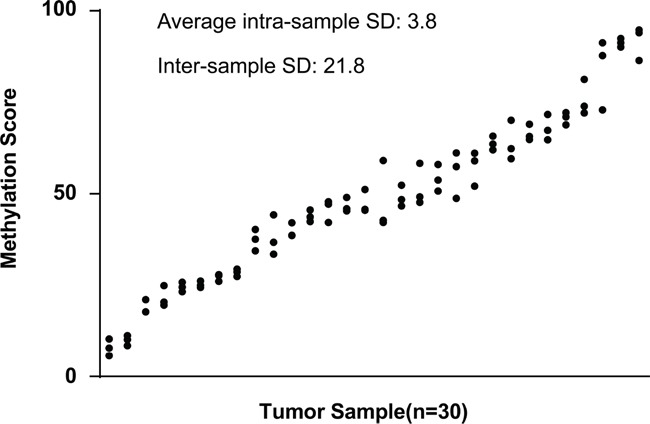
Intra- and inter-sample variability of methylation at the DAB2IP CpG1 DAB2IP CpG1 methylation score was determined in three different regions from the same tumor. Results from 30 different tumors, ranked by the mean methylation score for each tumor, were shown.

We further explored whether DAB2IP CpG1 methylation measurements may be affected by ITH by comparing results obtained from morphologically distinct regions of the same tumor. We collected tissues from three regions, representing the macroscopic heterogeneity of the tumors from 30 individuals. The interindividual differences, assessed by averaging all measurements from the same tumor, were significantly higher (standard deviation, 21.8%; range, 8.0% to 90.8%) than measurement differences within individual tumors (average standard deviation, 3.8%; range, 1.4% to 6.4%) (Figure 4). This result also suggests that the detection of DAB2IP CpG1 methylation was minimally affected by ITH in ccRCC.

### Correlation of DAB2IP CpG1 methylation with DAB2IP expression

**Figure 5:**
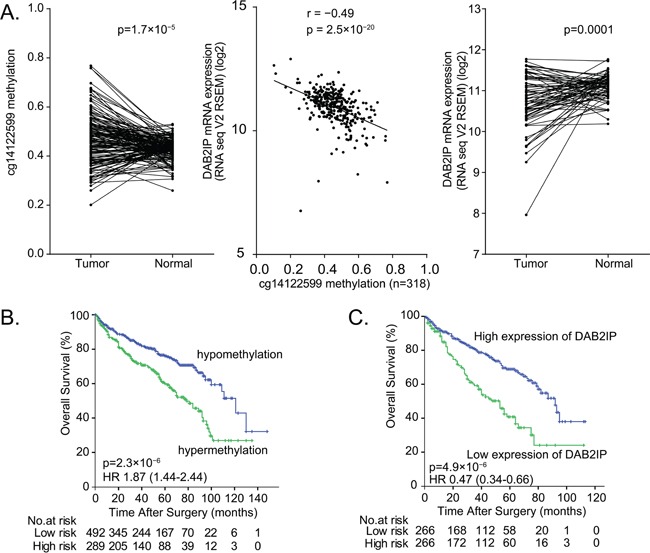
The prognosis value of DAB2IP CpG1 methylation and DAB2IP mRNA expression in ccRCC patients **A.** Left panel: Higher DAB2IP CpG1 methylation in tumor tissues compare to paired normal tissues (160 pairs) from TCGA set. Middle panel: Significant negative correlation between DAB2IP CpG1 methylation with DAB2IP mRNA expression in 318 patients from TCGA. Right panel: Lower mRNA expression of DAB2IP in tumor tissues compared to paired normal tissues. **B.** Hypermethylation of DAB2IP CpG1 correlates with poor survival of ccRCC patients. **C.** Low DAB2IP mRNA expression correlates with poor survival of ccRCC patients.

Using the TCGA dataset, our statistical analyses revealed a significantly increased DAB2IP CpG1 methylation in ccRCC compared to corresponding normal tissue (*n*=160 pairs, matched-pair t-test *p* = 1.7× 10^−5^; Figure 5A, left panel). We also analysed whether methylation of DAB2IP CpG1 was correlated with DAB2IP gene expression, per Spearman's correlation. We observed a significant inverse correlation between DAB2IP CpG1 methylation level and DAB2IP mRNA expression (r= −0.49, *p* = 2.5×10^−20^, Figure 5A, middle panel). Kaplan–Meier analysis based on the median cutoff value revealed that patients with high DAB2IP mRNA expression in ccRCC had an increased overall survival time compared to patients with low mRNA expression levels of the 532 patients in the TCGA dataset (log-rank test *p* =4.9×10^−6^, HR [95%CI]: 0.47 [0.34-0.66], Figure 5C).

### DAB2IP CpG1 methylation and DAB2IP mRNA expression after 5Aza-CdR treatment in 786-O and 769-P cells

**Figure 6:**
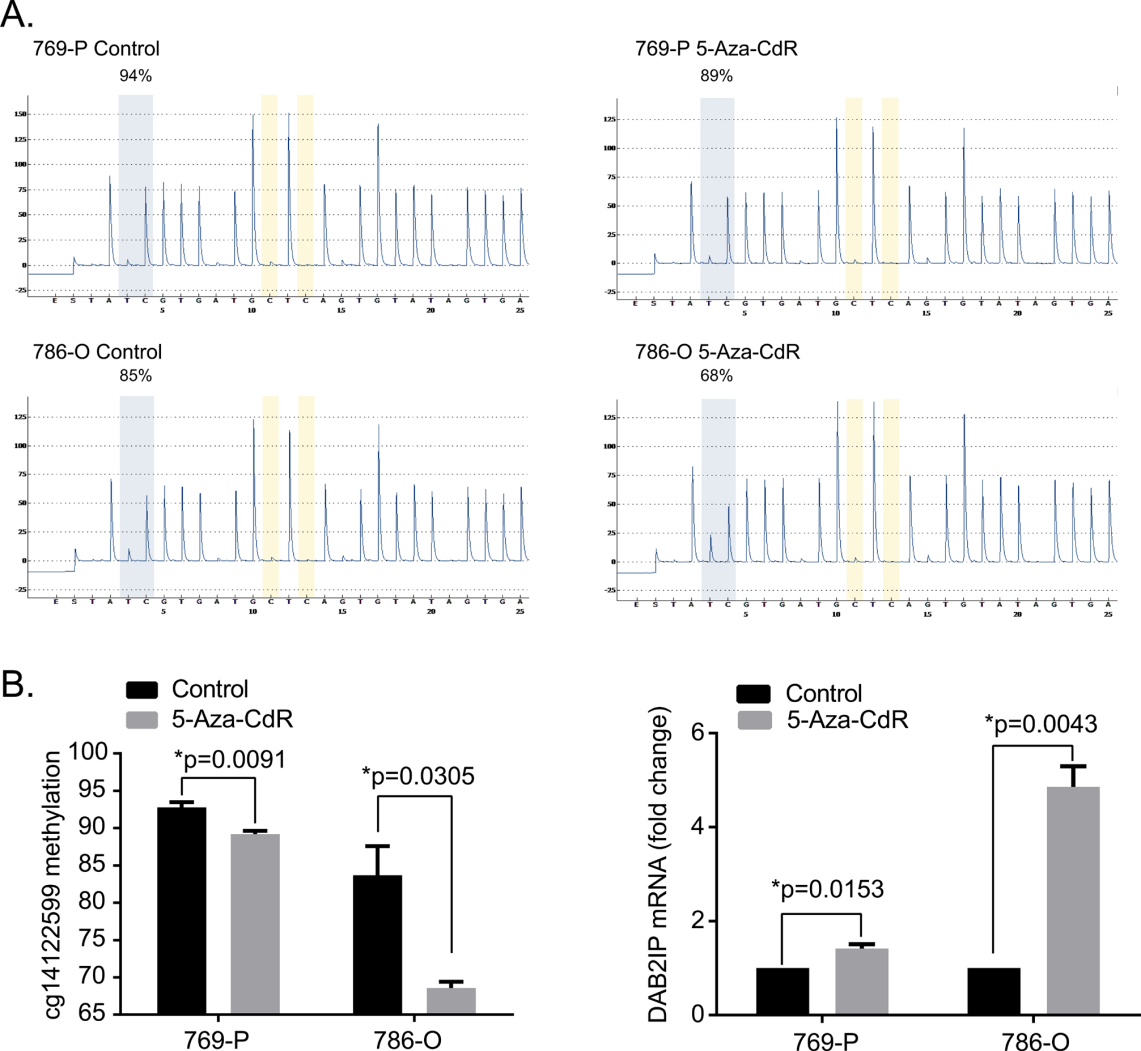
5-Aza-CdR treatment decrease DAB2IP CpG1 methylation and increase DAB2IP mRNA expression **A.** Representive pyrograms before and after 5-Aza-CdR treatment in 769-P and 786-O renal carcinoma cell lines **B.** 5-Aza-CdR treatment decrease DAB2IP CpG1 methylation and increase DAB2IP mRNA expression. Each data represented mean value ± standard deviation (SD, black error bars). *P* values were calculated using Student's t-test.

To assess the relationship of DAB2IP CpG1 methylation and DAB2IP mRNA expression in 786-O and 769-P RCC cells expressing low endogenous DAB2IP, we used DNA methyltransferase inhibitor (5-Aza-CdR) to treat the cells and detect the DAB2IP CpG1 methylation by pyrosequencing and DAB2IP mRNA expression by qPCR. After 5Aza-CdR treatment, DAB2IP CpG1 methylation level significantly decreased in 786-O cells, and 769-P human RCC cells (Student's t-test, both *p*<0.05). The decrease of DAB2IP CpG1 methylation level was accompanied by the significant increase of DAB2IP mRNA expression (Student's t-test, both *p*<0.05) (Figure 6).

## DISCUSSION

In the present study, we reported that DAB2IP CpG1 methylation in DAB2IP UTSS, the area important for transcriptional regulation, is associated with poorer survival in ccRCC patients. We validated these results from three independent series of ccRCC patients in TCGA, MCHC and UTSW. These findings suggest that DAB2IP CpG1 methylation can be a potential prognostic marker for ccRCC.

DAB2IP, a novel family of RasGTPase-activating protein family as a potent tumor suppressor, is epigenetically silenced [6, 14], which is suppressed by EZH2 and other epigenetic machinery such as DNA methylation and histone acetylation [15]. In this study, we identified methylation of DAB2IP CpG1 by pyrosequencing from FFPE material. DAB2IP CpG1 methylation can accurately distinguish between patients with ccRCC with substantially different clinical outcomes, even after adjustment for standard clinical prognostic factors, such as age, TNM stage, and Fuhrman grade. The association of DAB2IP CpG1 methylation and patient survival was observed not only from the discovery data set (TCGA) but also from two independent data sets (MCHC and UTSW) containing multiple hospitals. The association is apparently not dependent on the ethnicity of patients, which further supports that DAB2IP CpG1 methylation may serve as a repeatable biomarker in clinical practice.

Our data indicated that methylation status of DAB2IP CpG1 site was inversely correlated with DAB2IP expression. Also, overall methylation level of DAB2IP gene promoter is associated with the decreased expression of DAB2IP. Several studies suggest that some CpG sites more prone to methylation act as 'seeds' initializing hypermethylation and dictate gene expression [16–18]. The details molecular mechanisms of DAB2IP CpG1 methylation on gene expression remain unclear, further more studies are needed.

DNA methylation biomarkers can offer several advantages over genetic mutation markers [19, 20]. First, DNA methylation detection assay is highly sensitive and specific even from substantial contamination of normal DNA [21]. Second, epigenetic alterations may link life time environmental exposures with cancer risk, which can be used for evaluating risk factor of environmental exposures that are almost impossible to achieve based on genetic and environmental data alone [22]. Third, aberrant DNA methylation appears to occur in the early stage of tumor development, in contrast, gene mutation that occurs over years often requires sophisticated detection method [23]. Therefore, detection of aberrant DNA methylation is potentially good early indicator for risk assessment of cancer development. In addition, DNA methylation biomarkers also offer several advantages over mRNA markers. It is well established that mRNA levels are significantly altered by warm ischaemia times during RCC surgery [24]. Methylated DNA is far more stable than mRNA, does not require special handling requirements, can be easily detected retrospectively in archived samples, and is generally amenable to reliable analysis of patient samples [25, 26].

The analysis of multiple tumour regions from individual ccRCCs recently identified substantial ITH [27]. ITH can impair the precise molecular analysis of tumors because biomarker expression can vary across different tumor regions [28]. Extensive effort has been devoted to identifying molecular biomarkers for ccRCC [3, 29–31], but none of these studies have accounted for ITH. To further explore the impact of tumor heterogeneity on methylation analysis, we determined DAB2IP CpG1 methylation value of different samples from the same tumor tissue. In fact, we found relatively low variability of methylation measurements within individual tumor compared with the variability among different patient tumors, which further supports the potential of DAB2IP CpG1 methylation analysis for routine prognostic evaluation. Some other studies also showed DNA hypermethylation in the gene promoter regions seems to be less influenced by ITH [26, 32, 33], which are consistent with our results.

In summary, we identified and validated that DAB2IP CpG1 methylation is a practical prognostic biomarker for ccRCC that can add significant prognostic value to established clinicopathologic parameters. We validated DAB2IP CpG1 methylation as a prognostic maker in ccRCC patients from diverse geographic and racial backgrounds. DAB2IP CpG1 methylation was minimally affected by intratumoral heterogeneity in ccRCC.

## MATERIALS AND METHODS

### Patients

In this study, we used 463 formalin-fixed, paraffin-embedded (FFPE) tissue samples from 463 patients who underwent resection for ccRCC. The multiple Chinese centers (MCHC) set included 224 patients treated between 2001 and 2009 at three hospitals across different regions of China: First Affiliated Hospital of Sun Yat-sen University (Guangdong, southeast China), First Affiliated Hospital of Xi'an Jiaotong University (Shaanxi, northwest China), and Affiliated Hospital of Kunming University of Science and Technology (Yunnan, southwest China). Another 239 patients from University of Texas Southwestern Medical Center at Dallas (UTSW, TX, USA) treated between 2004 and 2011 comprised the UTSW set. The TNM 2009 staging system was used to classify ccRCC patients. The grading system used in the study was based on the Fuhrman four-grade. Additionally, intratumor heterogeneity (ITH) was investigated from morphologically distinct regions within the tumors of 30 patients with ccRCC treated between 2012 and 2014 at First Affiliated Hospital of Sun Yat-sen University (FFPE; three different regions coded as R1, R2, R3). The institutional review board at each participating institution approved retrospective analysis of anonymous patient data.

### TCGA data

For the TCGA set, clinical data, CpG methylation data (level 3 data, Infinium HumanMethylation450), and mRNA expression (level 3 data, RNA-seq Version 2 Illumina) were downloaded from the TCGA data portal (http://tcga-data.nci.nih.gov/tcga/) on Jun 1, 2015. The clinical data included 536 retrospectively identified patients who underwent radical or partial nephrectomy between 1998 and 2010 for sporadic ccRCC [31]. Of the 536 patients, *DAB2IP* CpG methylation data with the 450K array was available for 318 patients and *DAB2IP* mRNA expression data was available for 532 patients. There are three CpG sites of the *DAB2IP* gene located UTSS. We investigated the relationship between the three UTSS CpG methylation and patient prognosis.

### Pyrosequencing

The methylation level of CpG sites was evaluated with pyrosequencing in the MCHC, and UTSW sets. Genomic DNA was extracted with the QIAamp DNA FFPE Tissue Kit (Qiagen, Valencia, CA, USA) following the manufacturer's recommendations. Bisulfite conversion was performed on one microgram of DNA with the EpiTect Bisulfite Kit (Qiagen). Twenty nanograms of converted DNA were used as a template in each subsequent PCR. Specific sets of primers for PCR amplification and sequencing were designed using the PyroMark^®^ Assay Design 2.0 software (Qiagen). Primer sequences are listed as follow: Forward primer: GTTTTTTAGGGAGGGGTTT; Reverse primer: AAAAATAAAATAAAACAAACCTTAAACCTTAT; Sequencing Primer: TTGTTTTTTATGTTT. PCRs were performed with the PyroMark PCR Kit (Qiagen) under the following conditions: 95°C for 15 min; 45 cycles of 94°C for 30 sec, 56°C for 30 sec, and 72°C for 30 sec; and an elongation step of 72°C for 10 min. The success of amplification was assessed by 2% agarose gel electrophoresis. PCR products were pyrosequenced with the PyroMark Q24 pyrosequencer (Qiagen) according to the manufacturer's protocol (Pyro-Gold reagents). Output data were analyzed using PyroMark Q24 2.0.6 Software (Qiagen). Controls to assess proper bisulfite conversion of the DNA were included in each run, and sequencing controls were used to ensure the fidelity of the measurements.

### Intratumor heterogeneity (ITH)

ITH was investigated by extracting DNA samples from three morphologically distinct regions within the tumors of 30 patients with ccRCC treated between 2012 and 2014 at First Affiliated Hospital of Sun Yat-sen University (formalin-fixed paraffin-embedded specimens; three different regions coded as R1, R2, R3). Methylation of DAB2IP CpG1 was detected with pyrosequencing. Standard deviation (SD) was used to describe the inter-sample variability of CpG methylation between the 30 ccRCCs and the intra-sample variability between different regions.

### Cell cultures, 5-Aza-dC treatment, and qPCR

The human RCC cell lines (786-O, 769-P) were obtained from the American Type Culture Collection. The 786-O and 769-P lines were maintained in RPMI 1640 media contained 10% fetal bovine serum (FBS). Cells were incubated at 37°C at 5% CO2. For 5-Aza-2′-deoxycytidine (5-Aza-CdR) treatment, 3×10^4^ cells were seeded onto 60-mm dishes and treated with 5-Aza-dC at 10 μM for 4 days. 5-Aza-CdR was replenished daily during the treatment. At Day 4, 5-Aza-CdR was removed from the culture, and 5-Aza-CdR treated cells were washed with PBS and allowed to recover in normal culture medium. qPCR was performed as described in our previous reports [13]. All *in vitro* experiments were conducted in triplicate.

### Statistical analysis

Spearman's correlation coefficient was used to test the association between different variables. We used the Kaplan-Meier method to analyze the correlation between variables and overall survival, and we used the log-rank test to compare survival curves. Multivariate survival analysis was performed using the Cox regression model. Statistical analyses were carried out using IBM SPSS Statistics 20.0 (IBM, Armonk, NY). Statistical significance was set at 0.05.

## SUPPLEMENTARY FIGURES AND TABLE


