# Supplementary material for: Validation of DAB2IP methylation and its relative significance in predicting outcome in renal cell carcinoma
Source: Oncotarget. 2016 Apr 25;7(21):31508–19. doi: 10.18632/oncotarget.8971 (PMC5058774; doi:10.18632/oncotarget.8971)
Supplement: Supplementary file 1 [file oncotarget-07-31508-s001.pdf]

# Validation of DAB2IP methylation and its relative significance in predicting outcome in renal cell carcinoma

## SUPPLEMENTARY FIGURES AND TABLE

| Subgroup | No. of patients Total | Low risk (%) | High risk (%) | End−point | P value |
|---|---|---|---|---|---|
| All patients | 781 | 492 (63.0) | 289 (37.0) | 1.87 (1.44−2.44) | <0.0001 |
| Age | | | | | |
| <60 yr | 405 | 244 (60.2) | 161 (39.8) | 1.94 (1.27−2.98) | 0.002 |
| ≥60 yr | 376 | 248 (66.0) | 128 (34.0) | 1.98 (1.41−2.79) | <0.0001 |
| Sex | | | | | |
| Male | 505 | 367 (72.7) | 138 (27.3) | 2.27 (1.63−3.16) | <0.0001 |
| Female | 276 | 125 (45.3) | 151 (54.7) | 1.75 (1.07−2.84) | 0.025 |
| Race | | | | | |
| White | 445 | 289 (64.9) | 156 (35.1) | 1.63 (1.16−2.30) | 0.005 |
| Asian | 229 | 130 (56.8) | 99 (43.2) | 2.02 (1.25−3.29) | 0.004 |
| Black | 84 | 59 (70.2) | 25 (29.8) | 7.83 (2.43−25.18) | 0.001 |
| Grade | | | | | |
| G1/2 | 397 | 262 (66.0) | 135 (34.0) | 2.05 (1.26−3.36) | 0.004 |
| G3/4 | 380 | 227 (59.7) | 153 (40.3) | 1.69 (1.23−2.32) | 0.001 |
| Stage | | | | | |
| T1/2 | 531 | 351 (66.1) | 180 (33.9) | 1.96 (1.30−2.97) | 0.001 |
| T3/4 | 250 | 141 (56.4) | 109 (43.6) | 1.46 (1.04−2.07) | 0.031 |

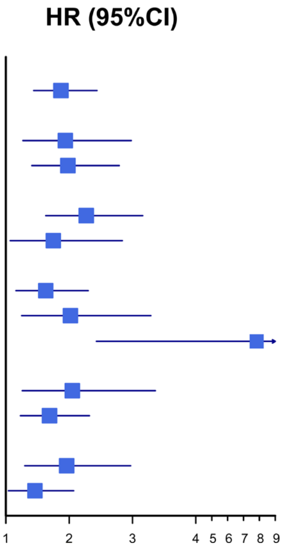


**Supplementary Figure S1: Hazard ratio of overall mortality for all 781 patients with ccRCC according to the methylation of the DAB2IP CpG1 in different subgroups stratified by clinical parameters.**

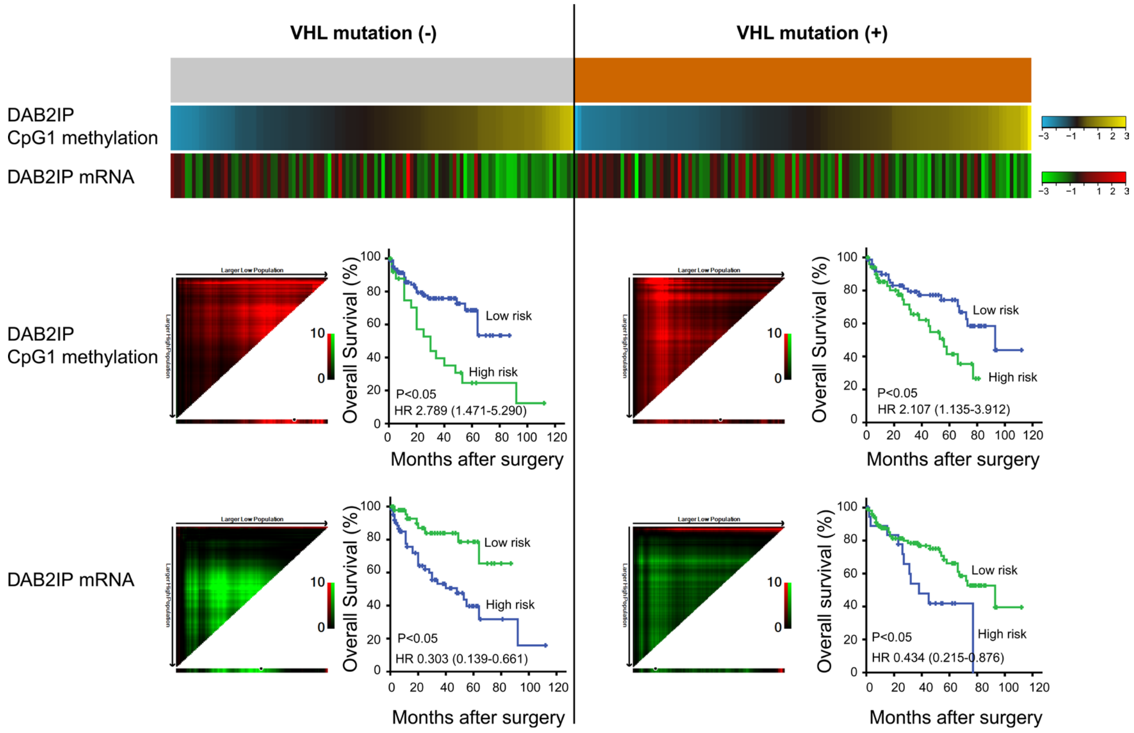


**Supplementary Figure S2: DAB2IP CpG1 methylation and DAB2IP mRNA in subsets of different VHL mutation status.** Based on the TCGA dataset analysis, DAB2IP CpG1 methylation and DAB2IP mRNA successfully predicted the prognosis of ccRCC in both VHL mutation (+) subtype and VHL mutation (-) subtype (all $p<0.05$). Patients in each subset were separated into high-risk and low-risk groups using X-tile plots to generate the optimum cut-off.

**Supplementary Table S1: 3 Probes located in UTSS of DAB2IP by the annotations provided by Illumina for the HumanMethylation450 platform**

| ID | CHR | MAPINFO | UCSC_RefGene_Name | UCSC_RefGene_Group |
|---|---|---|---|---|
| cg01305539 | 9 | 124328327 | DAB2IP | TSS1500 |
| cg14122599 | 9 | 124503625 | DAB2IP | TSS1500 |
| cg14383549 | 9 | 124503795 | DAB2IP | TSS1500 |
